# Long-term effects of neoadjuvant chemoradiotherapy followed by sphincter-preserving resection on anal sphincter function in relation to quality of life among locally advanced rectal cancer patients: a cross-sectional analysis

**DOI:** 10.1186/s13014-015-0479-4

**Published:** 2015-08-12

**Authors:** Zerrin Ozgen, Sevgi Ozden, Beste M Atasoy, Hazan Ozyurt, Rasim Gencosmanoglu, Nese Imeryuz

**Affiliations:** Clinic of Radiation Oncology, Marmara University Pendik Training and Research Hospital, Fevzi Cakmak Mah. Muhsin Yazicioglu Cad. No:10, 34899 Pendik, Istanbul Turkey; Clinic of Radiation Oncology, Dr. Lutfi Kirdar Training and Research Hospital, Istanbul, Turkey; Department of Radiation Oncology, Marmara University Faculty of Medicine, Istanbul, Turkey; Department of General Surgery, Marmara University Faculty of Medicine, Istanbul, Turkey; Department of Internal Medicine, Marmara University Faculty of Medicine, Istanbul, Turkey; Marmara University Gastroenterology Institute, Istanbul, Turkey

## Abstract

**Background:**

There is growing recognition for the consequences of rectal cancer treatment to maintain an adequate functional sphincter in the long-term rather than preserving the anal sphincter itself. This study aims to evaluate long-term effects of neoadjuvant chemoradiotherapy (nCRT) followed by sphincter-preserving resection on anal sphincter function in relation to quality of life (QoL) among locally advanced rectal cancer patients.

**Methods:**

Twenty-nine patients treated with nCRT followed by low anterior resection surgery were included in this study. Data on patient demographics, tumor location and symptoms of urgency and fecal soiling were recorded and evaluated with respect to Wexner Fecal Incontinence Scoring Scale, European Organization for Research and Cancer (EORTC) cancer-specific (EORTC QLQ-C30) and colorectal cancer-specific (EORTC QLQ-CR38) questionnaires and anorectal manometrical findings. Correlation of manometrical findings with Wexner Scale, EORTC QLQ-CR38 scores and EORTC QLQ-C30 scores was also evaluated.

**Results:**

Median follow-up was 45.6 months (ranged 7.5–98 months. Higher scores for incontinence for gas (*p* = 0.001), liquid (*p* = 0.048) and solid (*p* = 0.019) stool, need to wear pad (*p* = 0.001) and alteration in life style (*p* = 0.004) in Wexner scale, while lower scores for future perspective (*p* = 0.010) and higher scores for defecation problems (*p* = 0.001) in EORTC QLQ-CR38 were noted in patients with than without urgency. Manometrical findings of resting pressure (mmHg) was positively correlated with body image (r = 0.435, *p* = 0.030) and sexual functioning (r = 0.479, *p* = 0.011) items of functional scale, while rectal sensory threshold (RST) volume (mL) was positively correlated with defecation problems (r = 0.424, *p* = 0.031) items of symptom scale in EORTC QLQ-CR38 and negatively correlated with social function domain (r = −0.479, *p* = 0.024) in EORTC QLQ-C30. RST volume was also positively correlated with Wexner scores including incontinence for liquid stool (r = 0.459, *p* = 0.024), need to wear pad (r = 0.466, *p* = 0.022) and alteration in lifestyle (r = 0.425, *p* = 0.038).

**Conclusion:**

The high risk of developing functional anal impairment as well as the systematic registration of not only oncological but also functional and QoL related outcomes seem important in rectal cancer patients in the long-term disease follow-up.

## Background

Improved neoadjuvant chemoradiotherapy (nCRT) scheduling and planning have been associated with well-documented oncological benefits including down-staging and downsizing of the tumor leading increased tumor resectability and thus the higher likelihood of sphincter-preserving surgery [1–4]. Combined with advancement of surgical techniques, substantial improvement occurred in survival from rectal cancer over the past two decades [5–8]. This translated not only to a larger population of rectal cancer ‘survivors’ but also increased proportion of number of rectal cancer patients who can maintain the continuity of the intestine due to more frequent use of sphincter-preserving surgery by means of increased usage of nCRT, improved surgical technique and stapling devices [3, 5, 6].

However, while the greater use of neoadjuvant treatment and surgical techniques and thus survival from rectal cancer continues to improve, long-term consequences of treatment become unfolding. Consequently a growing body of evidence indicate that both preoperative radiotherapy and sphincter-preserving resection are likely to impair anorectal and sexual functions leading persistent dysfunctional symptoms which may have the potential to significantly impact on quality of life (QoL) [9–12], even more than a permanent stoma after abdominoperineal excision (APE) [13, 14].

As length of survival increases after diagnosis there is growing recognition for the consequences of cancer treatment to support patient recovery and optimize health-related QoL and thus for the importance of maintaining an adequate functional sphincter in the long-term rather than preserving the anal sphincter itself [3, 15].

Therefore, the present study was designed to evaluate long-term effects of nCRT followed by sphincter-preserving resection on anal sphincter function in relation to quality of life among locally advanced rectal cancer patients.

## Methods

### Study population

Of 74 patients with locally advanced (T3/T4 or N+) rectal adenocarcinoma who received nCRT prior to low anterior resection surgery between 2006 and 2013 and followed without any clinical or radiological disease progression, metastasis or recurrence thereafter, 29 patients (mean ± SD age: 54.7 ± 12.6 years, 51.7 % were males) were included in the study. None of patients had fecal soiling and thus complaints indicating presence of anal sphincter dysfunction at the time of diagnosis.

Written informed consent was obtained from each subject following a detailed explanation of the objectives and protocol of the study which was conducted in accordance with the ethical principles stated in the “Declaration of Helsinki” and approved by Marmara University School of Medicine Ethical Committee (reference no: 09.2011.0162, date: 24/11/2011).

### Study parameters

Data on patient demographics, tumor characteristics (preoperative stage, location, histological type, histological grade, postoperative stage, vascular invasion, and perineural invasion), treatment (nCRT, adjuvant chemotherapy) and treatment response (pathological) and symptoms of urgency and fecal soiling were recorded. Fecal incontinence was evaluated based on Wexner Fecal Incontinence Scoring Scale. Manometrical assessment of anal sphincter function was performed using anorectal manometry. Health-related quality of life (HRQoL) was evaluated with European Organization for Research and Cancer (EORTC) cancer-specific (EORTC QLQ-C30) and colorectal cancer-specific (EORTC QLQ-CR38) QoL questionnaires (QLQ). Manometrical findings of anal sphincter function, Wexner Fecal Incontinence Scoring Scale findings, EORTC QLQ-CR38 scores and EORTC QLQ-C30 scores were evaluated with respect to tumor location, gender and symptoms of urgency and fecal soiling. Correlation of manometrical findings with Wexner anal incontinence scores, EORTC QLQ-CR38 scores and EORTC QLQ-C30 scores was also evaluated.

### Chemoradiotherapy and surgery

Computed tomography (CT) simulation and 3D conformal treatment planning were performed in all patients. Radiotherapy was given with high-energy (15 or 18 MV photon) linear accelerator with 1.8 Gy per fraction to a total dose of 45–50.4 Gy in 25–28 fractions. During the whole CRT period all patients received 5-fluorouracil-based chemotherapy (400 mg/m^2^, i.v. bolus, at 1st and 5th week of radiotherapy or fluoropyrimidine 300 mg/m^2^/day, oral). Patients entered the study following the sphincter preserving surgery (low anterior resection) which was performed 6–8 weeks after nCRT and all had transient loop ileostomy and stoma reversal.

### Wexner fecal incontinence scoring scale

The Wexner Fecal Incontinence Scoring Scale [16] has become a widely used for the assessment of severity of fecal incontinence and has been validated to Turkish population, previously [17]. This scale included five questions that were three about anal incontinence (gas, liquid, and solid), a coping mechanism (pad wear), and a lifestyle question (alteration). Volunteers were instructed to rate the frequency of stool loss, frequency of use of coping behavior, and frequency of lifestyle alteration through the use of quantifiers (0 = never, 1 = rarely, 2 = sometimes, 3 = usually, 4 = always). The score was calculated by totaling the numerical values associated with the quantifiers. It provides a single anal incontinence severity score and higher scores indicate the severity of symptoms. The total score of the instrument ranged from 0 (no incontinence) to 20 (complete incontinence).

### Anorectal manometry

Anal sphincter manometry was performed median 17.3 months (mean 30 months, ranged 3–86 months) after the restorative surgery. Manometry was carried out by a pre-calibrated, water perfusion system (Mui Scientific Ontorio Canada model PIP-4-8SS), using a single use catheter with 8 holes 0.5 cm apart (Sandhill Scientific, CO, USA, SUAMC-M83A-10). Manometrical data was stored and analyzed with computer software (Sandhill Scientific Insight g3 Highlands Ranch, Colorado, USA). Bowel was prepared by enema (Fleet Enema, C.B. Fleet Co. Inc. USA). Patients were placed comfortably in the left lateral position. After the insertion of the catheter into the rectum, maximal resting pressure (MRP) and maximal squeeze pressure (MSP) measurements were done. MRP was defined as the average of maximal pressures obtained from all holes by stationary pull-through technique. MSP was defined as the mean of differences between the mean pressures obtained by voluntary squeezing for 5 s and resting pressures when all of the holes were within the anal canal. Patients were instructed to squeeze their anus as much as they could without squeezing their abdominal and buttock muscles throughout the entire period of MSP measurement in order to record actual sphincter pressures and to prevent pressure interactions. Minimum balloon volume that can be sensed was defined as rectal sensory threshold (RST) and minimal balloon distention volume that causes anal sphincter relaxation was defined as minimal balloon distention volume causing rectoanal inhibitory reflex (RAIR). We could not measure RAIR volume in 9 patients due to occurrence of anal pain.

### EORTC QLQ-C30

The EORTC QLQ-C30 is a 30-item general cancer instrument that evaluates 5 domains of QoL (physical, role, cognitive, emotional, and social), 9 symptom scales (fatigue, nausea/vomiting, pain, dyspnea, sleep disturbance, appetite loss, constipation, diarrhea, and financial impact), and a global QOL score. Each domain of QoL is assessed by 2–5 questions, and responses are scored on a 4-point Likert scale, with higher scores representing better QoL, whereas higher scores on symptom items represent worse symptoms. The validity and reliability of the QLQ-C30 has been well documented [18]. The reliability and validity of the Turkish version of QLQ-C30 has been confirmed [19].

### EORTC QLQ-CR38

The 38-item QLQ-CR38 is a colorectal cancer specific instrument that includes 4 functional domains (body image, sexual functioning, sexual enjoyment, and future perspective) and 8 symptom scales (micturition problems, chemotherapy side effects, symptoms associated with the gastrointestinal tract, male sexual problems, female sexual problems, defecation problems, stoma-related problems, and weight loss) [20]. Higher functional domain scores indicated increased function, and higher symptom scores signified more severe symptoms.

### Statistical analysis

Analyses were done in MedCalc Statistical Software version 12.7.7 (MedCalc Software bvba, Ostend, Belgium; http://www.medcalc.org; 2013). Median follow-up was calculated from the initiation of CRT. Manometrical scores of groups were compared using Mann–Whitney *U* test. Associations between parameters of Wexner scores, QoL scales, manometrical scores and complaints of patients (urgency or soiling) were assessed with Spearman test. Continuous variables are presented as mean ± SD or median (range) according to distribution of data *p* < 0.05 were considered statistically significant.

## Results and discussion

### Demographic and clinical characteristics of patients

Demographic and clinical characteristics of patients are summarized in Table 1. Median follow-up was 45.6 months (ranged 7.5–98 months). Most of patients had preoperative T3N1 (62.1 %) stage, distal (48.3 %) or mid-rectal (37.9 %), high grade (69.0 %) adenocarcinoma.

**Table 1:** Demographic and clinical characteristics of patients (*n* = 29)

Age (year), mean ± SD / median (min-max)	54.7 ± 12.6 / 53 (30–76)
Gender, n (%)	
Female	14 (48.3)
Male	15 (51.7)
Preoperative stage, n (%)	
T3N0	7 (24.1)
T3N1	18 (62.1)
T4N0	1 (3.4)
T2N0	2 (6.9)
T2N1	1 (3.4)
Location, n (%)	
Distal rectum	14 (48.3)
Mid-rectal	11 (37.9)
Proximal rectum	4 (13.8)
RT dose, n (%)	
50.4 Gy	18 (62.1)
56 Gy	7 (24.1)
46 Gy	4 (13.8)
Concomitant CT, n (%)	
FUFA Mayo	25 (86)
UFT+Antrex	4 (13.8)
CRT related complications, n (%)	
None	12 (41.4)
Grade 1	7 (24.1)
Grade 2–3	10 (34.5)
Histological grade, n (%)	
Low	7 (24.0)
High	20 (69.0)
Unknown	2 (7.0)
Postoperative N stage, n (%)	
N0	15 (93.8)
N1	13 (44.8)
N2	1 (6.3)
Pathological response, n (%) *n* = 29	
Complete response	7 (24.0)
Stable	10 (35.0)
Partial regression	12 (41.0)
Vascular invasion, n (%) *n* = 27	8 (29.6)
Perineural invasion, n (%) *n* = 27	6 (22.2)
Chemotherapy protocol, n (%) *n* = 21	
FUFA	16 (76.2)
XELOX	1 (4.8)
UFT+Antrex	3 (14.3)
FOLFOX	1 (4.8)
Urgency, n (%)	21 (77.0)
Fecal soiling, n (%)	9 (31.0)

*XELOX* Capacitabine and oxaliplatin, *FOLFOX* Fluorouracil and oxaliplatin

nCRT was applied using three dimensional linear accelerator radiotherapy in all patients, at 50.4 Gy dose in 62.1 % of patients and with FUFA Mayo chemotherapy regimen in 86 %. Two cT2N0 patients in our study received nCRT since they did not want to have APR surgery CRT related complications were observed in 58.6 % (Grade 1 in 24.1 % and Grade 2–3 in 34.5 %) of patients. Being assessed in 11 patients based on Ryan tumor regression grade system [21], pathological response rate was 24.0 %. Symptom of urgency was determined in 77.0 %, while fecal soiling in 31 % of patients. Tumor was determined to be located at distal rectum in 14(48.3 %) patients, mid-rectum in 11(37.9 %) patients and proximal rectum in 4(13.8 %) patients.

### Manometrical findings

Overall, resting pressure was 38.7 ± 21.9 mmHg, MSP was 116.9 ± 59.7 mmHg and RST volume was 28.1 ± 18.8 mL. Except for significantly higher MSP in males than in females (138.1 ± 62.7 mmHg vs. 94.1 ± 48.7 mmHg, *p* = 0.023), no significant difference was noted in manometrical findings with respect to tumor location, gender and symptoms of urgency and fecal soiling (Table 2).

**Table 2:** Manometrical findings with respect to tumor location, gender and symptoms of urgency and fecal soiling

		Total	Tumor location		Gender		Urgency		Fecal soiling	
			Distal rectum	Mid-rectal	Female	Male	(+)	(−)	(+)	(−)
Resting pressure (mmHg)	n^a^	27	13	10	13	14	19	8	9	18
	Mean ± SD	38.7 ± 21.9	32.4 ± 14.0	43 ± 24.0	42.5 ± 26.7	35.1 ± 16.4	33.3 ± 15.6	51.5 ± 29.7	30.6 + 14.0	42.7 + 24.2
	Median (min-max)	33.0 (12–96)	26 (17–62)	41 (16–88)	33 (12–96)	25.5 (17–62)	26 (12–62)	42.5 (19–96)	24 (17–54)	36 (12–96)
	*p* value^b^	-	0.456		0.720		0.137		0.226	
Maximal squeeze pressure (mmHg)	n^a^	29	14	11	14	15	21	8	9	20
	Mean ± SD	116.9 ± 59.7	110.3 ± 56.0	125.3 ± 49.8	94.1 ± 48.7	138.1 ± 62.7	109.5 + 56.2	136.4 + 68.2	135.8 + 77.8	108.4 + 50.0
	Median (min-max)	103.0 (35–273)	95 (39–240)	117 (35–211)	83.5 (39–211)	131 (35–273)	103 (35–240)	101.5 (73–273)	105 (35–273)	95 (39–211)
	*p* value^b^	-	0.273		**0.023**		0.305		0.409	
RST volume (mL)	n^a^	26	11	11	13	13	18	8	8	18
	Mean ± SD	28.1 ± 18.8	27.3 ± 15.6	30.9 ± 24.7	22.3 ± 7.3	33.8 ± 24.7	30.6 + 21.5	22.5 + 8.9	25 + 13.1	29.4 + 21.0
	Median (min-max)	20.0 (10–100)	20 (10–60)	20 (10–100)	20 (10–30)	20 (10–100)	20 (10–100)	20 (10–40)	20 (10–50)	20 (10–100)
	*p* value^b^	-	0.945		0.418		0.387		0.571	
MBDV causing RAIR (mL)	n^a^	20	8	8	12	8	14	6	6	14
	Mean ± SD	27.5 ± 10.2	26.3 ± 7.4	27.5 ± 11.6	25 ± 6.7	31.3 ± 13.6	26.4 ± 9.3	30 ± 12.6	31.7 ± 11.7	25.7 ± 9.4
	Median (min-max)	20(20–50)	25(20–40)	25(20–50)	20(20–40)	25(20–50)	20(20–50)	25(20–50)	30(20–50)	20(20–50)
	*p* value^b^	-	0.959		0.427		0.659		0.274	

*RST* Rectal sensory threshold, *MBDV* Minimal balloon distension volume, *RAIR* Rectoanal inhibitory reflex

^a^Patients numbers in the table refers to patients with available data. ^b^Mann Whitney *U* test. Bold values indicate statistical significance at α=0.05 level

### Wexner fecal incontinence scores

Total Wexner score was median 10.0 with no difference in component scores with respect to tumor location, while a significant influence of gender and symptoms on incontinence scores. Scores of incontinence for solid stool (*p* = 0.029) and alteration in life style (*p* = 0.012) were significantly higher males than in females. In patients with than without urgency, significantly higher total scores (*p* < 0.001) and component scores of incontinence for gas (*p* = 0.001) and liquid (*p* = 0.048) and solid (*p* = 0.019) stool as well as for need to wear pad (*p* = 0.001) and alteration in life style (*p* = 0.004) were determined. In patients with than without fecal soiling, scores of incontinence for solid stool (*p* = 0.039) and alteration in life style (*p* = 0.034) were significantly higher (Table 3).

**Table 3:** Wexner Fecal Incontinence Scores with respect to tumor location, gender and symptoms of urgency and fecal soiling

	Total	Tumor location in rectum				Gender			Urgency			Fecal soiling		
		Prox.	Mid.	Distal		F	M		(+)	(−)		(+)	(−)	
Wexner scale components	Median (min-max)	Median (min-max)			P^a^	Median (min-max)		P^b^	Median (min-max)		P^b^	Median (min-max)		**p** ^b^
Incontinence for gas	4.0 (0–4)	2 (0–4)	4 (0–4)	4 (0–4)	0.662	3.5 (0–4)	4 (0–4)	0.458	4 (0–4)	0 (0–4)	**0.001**	4 (0–4)	4 (0–4)	0.658
Incontinence for liquid stool	0 (0–4)	0 (0–4)	0 (0–4)	2.5 (0–4)	0.753	0 (0–4)	3 (0–4)	0.169	3 (0–4)	0 (0–3)	**0.048**	0 (0–4)	0 (0–4)	0.418
Incontinence for solid stool	0 (0–4)	0 (0–4)	0 (0–4)	0.5 (0–4)	0.779	0 (0–4)	4 (0–4)	**0.029**	3 (0–4)	0 (0–0)	**0.019**	0 (0–4)	0 (0–4)	**0.039**
Need to wear pad	2 (0–4)	0.5 (0–4)	3 (0–4)	2.5 (0–4)	0.649	0.5 (0–4)	4 (0–4)	0.068	3.5 (0–4)	0 (0–1)	**0.001**	1 (0–4)	1 (0–4)	0.051
Alteration in lifestyle	2 (0–4)	0.5 (0–4)	3 (0–4)	2 (0–4)	0.689	0 (0–4)	4 (0–4)	**0.012**	3 (0–4)	0 (0–1)	**0.004**	0 (0–4)	0 (0–4)	**0.034**
Total score	10 (0–20)	3 (0–20)	11 (0–20)	10.5 (0–20)	0.690	4 (0–20)	16 (0–20)	0.061	14.5 (0–20)	0 (0–4)	**<0.001**	4 (0–20)	4 (0–20)	0.106

^a^Kruskal Wallis test, ^b^Mann-Whitney *U* test. Bold values indicate statistical significance at α=0.05 level

*F* female, *M* male

### Correlation of manometrical findings with Wexner Fecal Incontinence scores

RST volume was positively correlated with incontinence for liquid stool (r = 0.459, *p* = 0.024), need to wear pad (r = 0.466, *p* = 0.022) and alteration in lifestyle (r = 0.425, *p* = 0.038) components of Wexner scale. Minimal balloon distention volume causing RAIR was positively correlated with incontinence for liquid stool (r = 0.586, *p* = 0.008) (Table 4).

**Table 4:** Correlation of manometrical findings with Wexner Fecal Incontinence scores

	Manometrical findings							
	Resting pressure (mmHg)		MSP (mmHg)		RST volume (mL)		MBDV causing RAIR (mL)	
Wexner scores for:	r	p	r	p	r	p	r	p
Incontinence for gas	−0.280	0.165	−0.273	0.168	0.324	0.123	−0.022	0.928
Incontinence for liquid stool	0.095	0.646	−0.167	0.404	0.459	**0.024**	0.586	**0.008**
Incontinence for solid stool	−0.075	0.717	−0.111	0.582	0.396	0.056	0.373	0.116
Need to wear pad	−0.10	0.626	−0.121	0.546	0.466	**0.022**	0.351	0.141
Alteration in lifestyle	−0.033	0.873	0.126	0.531	0.425	**0.038**	0.291	0.226

*MSP* Maximal squeeze pressure, *RST* Rectal sensory threshold, *MBDV* Minimal balloon distension volume, *RAIR* Rectoanal inhibitory reflex, *r* Correlation coefficient. Bold values indicate statistical significance at α=0.05 level

Spearman correlation analysis

No significant difference was noted in Wexner as well as QoL scores with respect to minimal balloon distention volume causing or not causing RAIR (Table 5).

**Table 5:** Wexner scores with respect to minimal balloon distention volume (causing vs. not causing RAIR)

	Minimal balloon distention volume		
Med (min-max)	RAIR (−)	RAIR (+)	*p* value^a^
Wexner scores for:			
Incontinence for gas	6(1–6)	5(1–6)	0.238
Incontinence for liquid stool	4.5(1–6)	1(1–6)	0.260
Incontinence for solid stool	5.5(1–6)	1(1–6)	0.147
Need to wear pad	5.5(1–6)	3(1–6)	0.132
Alteration in lifestyle	6(1–6)	2(1–6)	0.084
Qol Score	50(25–83.3)	58.3(16.7–100)	0.982

^a^Mann Whitney *U* test

### EORTC QLQ-CR38 scores

Overall scores for functional and symptoms scales of EORTC QLQ-CR38 are shown in Table 6. Tumor location could not be evaluated. No significant difference was noted with respect to gender and symptoms of fecal soiling. Patients with than without urgency symptom have significantly lower scores for future perspective (*p* = 0.010) and sexual functioning (*p* = 0.016) items of functional scales, while higher scores for chemotherapy side effects (*p* = 0.023) and defecation problems (*p* = 0.001) items of symptoms scales (Table 6).

**Table 6:** EORTC QLQ-CR38 scores with respect to tumor location, gender and symptoms of urgency and fecal soiling

EORTC QLQ-CR38 components	Total	Tumor location in rectum				Gender			Urgency			Fecal soiling		
		Prox.	Mid.	Distal		F	M		(+)	(−)		(+)	(−)	
Functional scales	Median (min-max)	Median (min-max)			P^a^	Median (min-max)		P^a^	Median (min-max)		P^a^	Median (min-max)		**P** ^**a**^
Body image (*n* = 26)	77.8 (33.3–100)	88.9 (55.6–100)	66.7 (33.3–100)	94.4 (33.3–100)	NA	88.9 (33.3–100)	61.1 (33.3–100)	0.274	66.7 (33.3–100)	100 (33.3–100)	0.151	66.7 (33.3–100)	77.8 (33.3–100)	0.469
Future perspective (*n* = 25)	66.7 (0–100)	83.3 (66.7–100)	66.7 (0–100)	66.7 (0–100)	NA	66.7 (0–100)	66.7 (0–100)	0.727	66.7 (0–100)	100 (66.7–100)	**0.010**	66.7 (33.3–100)	66.7 (0–100)	0.855
Sexual functioning (*n* = 29)	33.3 (0–66.7)	8.3 (0–33.3)	33.3 (0–66.7)	33.3 (0–66.7)	NA	0 (0–66.7)	33.3 (0–66.7)	0.172	0 (0–66.7)	33.3 (16.7–66.7)	**0.016**	16.7 (0–50)	33.3 (0–66.7)	0.521
Sexual enjoyment (*n* = 24)	0 (0–66.7)	0 (0–33.3)	0 (0–33.3)	0 (0–66.7)	NA	0 (0–33.3)	0 (0–66.7)	0.977	0 (0–33.3)	0 (0–66.7)	0.634	0 (0–0)	0 (0–66.7)	0.273
Symptom scales														
Rad-induced micturition (*n* = 29)	22.2 (0–77.8)	16.7 (0–44.4)	22.2 (0–55.6)	27.8 (0–77.8)	NA	11.1 (0–77.8)	33.3 (0–66.7)	0.112	22.2 (0.7–7.8)	5.6 (0–66.7)	0.206	22.2 (11.1–66.7)	11.1 (0–77.8)	0.250
Chemotherapy side effects (*n* = 29)	11.1 (0–100)	27.8 (0–100)	11.1 (0–33.3)	11.1 (0–33.3)	NA	11.1 (0–100)	11.1 (0–33.3)	0.477	11.1 (0–100)	0 (0–22.2)	**0.023**	11.1 (0–22.2)	11.1 (0–100)	0.304
Gastrointesti-nal symptoms (*n* = 29)	20 (0–60)	30 (0–60)	6.7 (0–53.3)	20 (0–53.3)	NA	20 (0–60)	13.3 (0–53.3)	0.425	20 (0–60)	6.7 (0–26.7)	0.114	20 (0–53.3)	16.7 (0–60)	0.685
Sexual dysfunction of men (*n* = 11)	50 (0–100)	0 (0–0)	100 (33.3–100)	41.7 (16.7–100)	NA	–	50 (0–100)	NA	58.3 (16.7–100)	16.7 (0–100)	0.294	33.3 (0–100)	75 (16.7–100)	0.496
Sexual dysfunction of women (*n* = 4)	0 (0–50)	50 (50–50)	–	0 (0–0)	NA	50 (0–100)	–	NA	0 (0–0)	0 (0–50)	NA	**–**	0 (0–50)	0.113
Defecation problems (*n* = 29)	33.3 (0–85.7)	23.8 (4.8–42.9)	33.3 (0–81)	33.3 (4.8–85.7)	NA	30.9 (0–57.1)	33.3 (0–85.7)	0.425	38.1 (4.8–85.7)	7.1 (0–33.3)	**0.001**	38.1 (4.8–85.7)	30.9 (0–81)	0.502
Weight loss (*n* = 29)	0 (0–100)	0 (0–100)	0 (0–66.7)	0 (0–33.3)	N/A	0 (0–100)	0 (0–66.7)	0.621	0 (0–100)	0 (0–0)	0.097	0 (0–33.3)	0 (0–100)	1.00

^a^Mann-Whitney *U* test

*F* Female, *M* Male, *NA* Not applicable (*n* < 30), *Rad* Radiation. Bold values indicate statistical significance at α=0.05 level

### Correlation of manometrical findings with EORTC QLQ-CR38 scores

Resting pressure (mmHg) was positively correlated with body image (r = 0.435, *p* = 0.030) and sexual functioning (r = 0.479, *p* = 0.011) items of functional scale. RST volume was positively correlated with defecation problems (r = 0.424, *p* = 0.031) and weight loss (r = 0.445, *p* = 0.023) items of symptom scale in EORTC QLQ-CR38 (Table 7).

**Table 7:** Correlation of manometrical findings with EORTC QLQ-CR38 scores

	Manometrical findings							
	Resting pressure (mmHg)		MSP (mmHg)		RST volume (mL)		MBDV causing RAIR (mL)	
EORTC QLQ-CR38 components	r	p	r	p	r	p	r	p
Functional scales								
Body image (*n* = 26)	**0.435**	**0.030**	0.160	0.435	−0.173	0.420	0.059	0.810
Future perspective (*n* = 25)	0.081	0.714	0.189	0.365	−0.276	0.214	−0.024	0.931
Sexual functioning (*n* = 29)	**0.479**	**0.011**	0.268	0.160	−0.143	0.486	0.353	0.127
Sexual enjoyment (*n* = 24)	0.299	0.176	−0.049	0.821	0.091	0.694	0.005	0.985
Symptom scales								
Radiation-induced micturition (*n* = 29)	−0.263	0.186	0.015	0.937	−0.091	0.659	−0.411	0.072
Chemotherapy side effects (*n* = 29)	0.183	0.361	0.004	0.982	−0.203	0.319	−0.344	0.137
Gastrointestinal symptoms (*n* = 29)	−0.304	0.123	−0.232	0.226	0.025	0.904	0.185	0.435
Sexual dysfunction of men (*n* = 11)	−0.087	0.812	−0.304	0.364	0.464	0.176	0.268	0.254
Sexual dysfunction of women (*n* = 4)	0.775	0.225	−0.258	0.752	−0.577	0.423	0.232	0.325
Defecation problems (*n* = 29)	−0.159	0.429	−0.163	0.398	**0.424**	**0.031**	0.242	0.304
Weight loss (*n* = 29)	−0.146	0.468	−0.021	0.913	**0.445**	**0.023**	0.00	1.00

*MSP* Maximal squeeze pressure, *RST* Rectal sensory threshold, *MBDV* Minimal balloon distension volume, *RAIR* Rectoanal inhibitory reflex, *r* Correlation coefficient

Spearman correlation analysis. Bold values indicate statistical significance at α=0.05 level

### EORTC QLQ-C30 scores

Overall scores for functional and symptoms scales of EORTC QLQ-C30 are shown in Table 8. Tumor location could not be evaluated. No significant difference was noted with respect to gender. Global QoL/general health status (GHS) scores were significantly lower in patients with than without urgency (*p* = 0.039) or fecal soiling (9 = 0.008) symptoms. Role function scores of functional scale were significantly lower in patients with than without urgency (*p* = 0.020), while emotional function scores (*p* = 0.032) of functional scale and fatigue score (*p* = 0.043) of symptom scale were significantly lower in patients with than without fecal soiling (Table 8).

**Table 8:** EORTC QLQ-C30 scores with respect to tumor location, gender and symptoms of urgency and fecal soiling

	Total	Tumor location in rectum			Gender				Urgency			Fecal soiling		
		Prox.	Mid.	Distal		F	M		(+)	(−)		(+)	(−)	
	Median (min-max)	Median (min-max)			P^a^	Median (min-max)		P^a^	Median (min-max)		P^a^	Median (min-max)		**P** ^**a**^
EORTC QLQ-C30 components														
Global QoL/GHS	58.3 (16.7–100)	54.2 (33.3–66.7)	58.3 (16.7–83.3)	41.7 (16.7–100)	NA	58.3 (33.3–100)	41.7 (16.7–100)	0.331	41.7 (16.7–100)	70.8 (33.3–100)	**0.039**	33.3 (16.7–66.7)	66.7 (16.7–100)	**0.008**
Functional scales														
Physical function	86.7 (0–100)	73.3 (20–100)	93.3 (40–100)	83.3 (0–100)	NA	90 (0–100)	80 (40–100)	1.00	80 (20–100)	96.7 (0–100)	0.345	80 (53.3–100)	86.7 (0–100)	0.943
Role function	91.7 (33.3–100)	83.3 (33.3–100)	100 (66.7–100)	66.7 (33.3–100)	NA	100 (33.3–100)	83.3 (50–100)	0.776	83.3 (33.3–100)	100 (83.3–100)	**0.020**	66.7 (50–100)	100 (33.3–100)	0.495
Emotional function	75 (25–100)	70.8 (25–75)	79.2 (33.3–100)	75 (41.7–100)	NA	75 (25–100)	75 (33.3–100)	0.571	70.8 (25–100)	87.5 (50–100)	0.136	66.7 (33.3–91.7)	83.3 (25–100)	**0.032**
Cognitive function	83.3 (50–100)	83.4 (66.7–100)	83.3 (50–100)	91.7 (50–100)	NA	83.3 (50–100)	83.3 (50–100)	0.561	83.3 (50–100)	91.7 (50–100)	0.491	100 (66.7–100)	83.3 (25–100)	0.554
Social function	66.7 (16.7–100)	66.7 (50–83.3)	66.7 (16.7–100)	66.7 (33.3–100)	NA	83.3 (33.3–100)	50 (16.7–100)	0.320	50 (16.7–100)	83.3 (33.3–100)	0.228	50 (33.3–83.3)	75 (16.7–100)	0.370
Symptom scales														
Fatigue	22.2 (11.1–77.8)	55.6 (11–1)	22.2 (11.1–44.4)	22.2 (11.1–77.8)	NA	22.2 (11.1–77.8)	33.3 (11.1–55.6)	0.683	33.3 (11.1–77.8)	11.1 (11.1–55.6)	0.162	11.1 (11.1–44.4)	38.9 (11.1–77.8)	**0.043**
Nausea / vomiting	0 (0–66.7)	8.3 (0–33.3)	0 (0–66.7)	0 (0–66.7)	NA	0 (0–66.7)	0 (0–66.7)	0.780	0 (0–66.7)	0 (0–66.7)	0.820	0 (0–66.7)	0 (0–66.7)	1.00
Pain	0 (0–100)	16.7 (0–33.3)	0 (0–100)	0 (0–100)	NA	8.3 (0–100)	0 (0–100)	0.591	0 (0–100)	0 (0–100)	0.936	0 (0–100)	0 (0–100)	0.776
Dyspnea	0 (0–33.3)	0 (0–0)	0 (0–0)	0 (0–33.3)	NA	0 (0–33.3)	0 (0–33.3)	0.780	0 (0–100)	0 (0–33.3)	0.817	0 (0–33.3)	0 (0–33.3)	0.166
Sleep disturbance	0 (0–100)	66.7 (0–100)	33.3 (0–100)	0 (0–100)	NA	0 (0–100)	33.3 (0–100)	0.505	33.3 (0–100)	0 (0–66.7)	0.185	33.3 (0–100)	0 (0–100)	0.522
Loss of appetite	0 (0–100)	16.7 (0–100)	0 (0–33.3)	0 (0–66.7)	NA	0 (0–100)	0 (0–33.3)	0.621	0 (0–100)	0 (0–66.7)	0.896	0 (0–33.3)	0 (0–100)	0.256
Constipation	0 (0–100)	33.3 (0–100)	0 (0–33.3)	0 (0–100)	NA	0 (0–100)	0 (0–33.3)	0.747	0 (0–100)	0 (0–100)	0.436	0 (0–33.3)	0 (0–100)	0.278
Diarrhea	0 (0–100)	16.7 (0–33.3)	0 (0–100)	0 (0–100)	NA	0 (0–100)	0 (0–100)	0.813	0 (0–100)	33.3 (0–100)	0.126	0 (0–66.7)	0 (0–100)	0.701
Financial impact	0 (0–100)	33.3 (0–100)	0 (0–100)	50 (0–100)	NA	16.7 (0–100)	33.3 (0.0–100)	0.747	33.3 (0–100)	0 (0–100)	0.423	66.7 (0–100)	0 (0–100)	**0.234**

^a^Mann-Whitney *U* test

*F* Female, *M* Male, *NA* Not applicable (*n* < 30), *Rad* Radiation. Bold values indicate statistical significance at α=0.05 level

### Correlation of manometrical findings with EORTC QLQ-C30 scores

Except for significant negative correlation of social function scores of functional scales to RST volume (r = −0.479, *p* = 0.024), no correlation was noted between EORTC QLQ-C30 scores and manometrical findings (Table 9).

**Table 9:** Correlation of manometrical findings with EORTC QLQ-C30 scores

	Manometrical findings					
	Resting pressure (mmHg)		MSP (mmHg)		RST volume (mL)	
EORTC QLQ-C30 components	r	p	r	p	r	p
Global QoL/GHS	0.188	0.347	−0.072	0.711	−0.070	0.734
Functional scales						
Physical function	0.090	0.657	−0.046	0.813	−0.0257	0.205
Role function	0.096	0.662	0.194	0.363	−0.092	0.683
Emotional function	0.276	0.163	−0.056	0.778	−0.043	0.839
Cognitive function	−0.004	0.986	−0.185	0.337	−0.095	0.646
Social function	0.366	0.086	0.070	0.741	**−0.479**	**0.024**
Symptom scales						
Fatigue	0.235	0.248	−0.042	0.834	−0.080	0.710
Nausea/vomiting	−0.208	0.299	−0.141	0.467	−0.135	0.510
Pain	−0.305	0.122	−0.054	0.779	0.181	0.377
Dyspnea	−0.250	0.209	0.163	0.400	0.215	0.290
Sleep disturbance	−0.265	0.182	0.030	0.876	0.254	0.210
Loss of appetite	−0.108	0.592	−0.107	0.581	−0.097	0.638
Constipation	−0.034	0.866	0.239	0.211	−0.025	0.902
Diarrhea	0.292	0.139	0.319	0.092	−0.042	0.840
Financial impact	−0.066	0.745	0.076	0.696	0.202	0.323

*MSP* Maximal squeeze pressure, *RST* Rectal sensory threshold, *r* correlation coefficient

Spearman correlation analysis. Bold values indicate statistical significance at α=0.05 level

In the present cohort of locally advanced rectal cancer patients treated with nCRT and sphincter-preserving resection, after a median follow up of 45.6 months, anorectal manometry performed after median 17.3 months of restoration revealed an average resting pressure (38.7 mmHg) and MSP (116.9 mmHg) of below the normal ranges (60–80 mmHg and >120 mmHg, respectively) [22]. Average Wexner score (10.0) was consistent with severe fecal incontinence (scores ≥10) [22, 23], while GHS scores were suggestive of poor HRQoL. Neither the severity of incontinence nor the manometrical findings differed with respect to tumor location, while the presence of urgency and impaired RST volume were associated with increased severity of fecal incontinence and poor QoL scores.

Pelvic dysfunction including bowel, urinary and sexual dysfunctions has been reported in up to 25–50 % of all patients following a sphincter-preserving resection of the rectum [24–26] as a result of reduced anal tone, loss of recto-anal inhibitory reflex, and decreased neorectal volume [5]. nCRT has also been documented to lead to adverse effects on bowel function based on vascular toxicity and direct damage to the anal sphincter muscle leading decrease in anal resting pressure and colonic compliance and inhibition of the impulse conduction [9, 11].

Hence, both pelvic radiotherapy and sphincter-preserving surgery are considered likely to impair anorectal and sexual functions and thereby to lead poor QoL in rectal cancer patients [5, 9, 11, 12]. Accordingly our findings support the emerging literature on late adverse effects of pre-operative radiotherapy and sphincter-preserving surgery on bowel function in rectal cancer patients with restoration of bowel continuity as associated with increased frequency, urgency, incomplete rectal emptying and fecal leakage/incontinence problems [9, 27–30] along with a significant impact on QoL [29, 31, 32].

Bowel dysfunction is considered to be most frequent and severe within the first year of treatment, and then to stabilize in general [5], while longer persistence of the problem extending to 5 years postoperatively has also been reported in 5–63 % of rectal cancer patients [5, 33, 34].

Accordingly, albeit it is not possible to discriminate the impact of surgery or nCRT *per se* on the bowel dysfunction, identification of decreased resting pressure MSP, severe fecal incontinence and presence of urgency in most of our patients after a median follow up of 45.6 months supports that a considerable proportion of patients with rectal cancer suffer from bowel dysfunction for 3–5 years [29, 35–37].

Data on the prevalence of incontinence and urgency aspects of bowel dysfunction following low anterior resection revealed large variations in different studies, ranging from 0 to 51 % and 4 to 68 % respectively [38]. Fecal incontinence is considered to be a common problem among patients with rectal cancer after surgery, recovered over time [39–41] in some patients, while as in our cohort it may also remain as a long-term problem in some patients with rectal cancer [5].

Identification of urgency in 77.0 % of our cohort supports the consideration of urgency as a common defecation related problem among patients with rectal cancer in the first 5 years following surgery, experienced by 40–46 % of patients at year four [29, 37, 40], and 16–38 % at year five [29, 42]. The significant association of presence of urgency with severity of all component scores of Wexner fecal incontinence scale in our cohort supports the association of the alteration in sensation of defecation after resection with development of incontinence in operated rectal cancer patients [43].

Presence of fecal soiling in 30 % of our patients support that fecal incontinence and consequent loss of control over defecation are also associated with other problems such as fecal leaking/soiling among patients with fecal incontinence as reported in the past studies with a prevalence ranging from 10 to 69 % during the first 3 years after surgery [5, 31, 35, 44–46].

In a past study on prospective evaluation of bowel function after sphincter-preserving surgery for rectal cancer, decrease in bowel function scores was reported in 163/266 patients (61.3 %) within the first postoperative year, while the tumor location was reported to be independently associated with impaired bowel function in the multivariable analysis [47].

In another study considering the short-term preoperative change of anorectal function based on manometric data after nCRT, tumor response of chemoradiation was shown to be significantly associated with sensory threshold for desire to defecate and thus potential benefit of nCRT on defecatory function and local disease control has been suggested, at least in the short-term period after the radiation [48].

However, no difference was noted in manometrical findings, Wexner scores and cancer specific and colorectal cancer specific QoL measures in the long-term with respect to tumor location in our cohort of rectal cancer patients. But it is quite likely that having relatively small sample size might preclude achieving statistical significance in these parameters.

Bowel dysfunction, leading lifestyle changes in 33–58 % of patients at year one, has been shown to have a profound effect on daily life and the QoL of patients with rectal cancer [46, 49]. Accordingly, GHS scores were suggestive of poor QoL in our cohort. Sexual functioning and sexual enjoyment were the mostly affected functional domains and sexual dysfunction of men and defecation problems were the most severe symptoms identified on EORTC QLQ-CR38 in our patients. Likewise, recent research has shown high levels of sexual dysfunction in males undergoing rectal cancer treatment [6, 11, 28]. However it should be noted the EORTC QLQ-CR38 only measures sexual interest and enjoyment and not the impact of psychological factors on sexual functioning which seems an important component of female sexual dysfunction [6, 50].

Patients with rectal cancer have to adapt themselves behaviorally and psychologically to manage bowel dysfunction via lifestyle changes such as taking drugs, using pads, modifying diet, and reducing social activities to avoid occurrence of bowel accidents in public places [13, 51]. Accordingly, social function was the mostly affected functional domain and financial impact was amongst the most severe symptoms in EORTC QLQ-C30 in our cohort which seems consistent with the inverse association reported between bowel dysfunction and social functioning [29, 52] and the higher likelihood of financial difficulties in patients reporting greater defecation problems [6, 29] reported in the past studies.

Although fecal incontinence after radiotherapy has been reported to affect QoL of rectal cancer patients in significant and persistent manner [53], urgency and clustering were suggested to have a much higher impact on QoL than fecal incontinence [38]. A statistically significant association between urgency and incomplete emptying and QoL, but not between incontinence and QoL was also reported in rectal cancer patients [54].

Notably, in addition to its significant association with severity of fecal incontinence, urgency was also associated with poorer function in future perspective and sexual functioning domains of EORTC QLQ-CR38 along with increased severity of chemotherapy related side effects and defecation problems in our cohort. Presence of urgency and fecal soiling symptoms were also associated with poor GHS scores along with poor role function and emotional function, respectively on EORTC QLQ-C30.

With no significant difference in terms of manometrical findings, severity of fecal incontinence and poor colorectal cancer-specific QoL were more pronounced in patients with than without urgency in our cohort. Indeed, impaired RST volume and presence of urgency were the two parameters that showed the highest positive association with Wexner fecal incontinence scores. Besides, RST volume was correlated negatively with social function domain of EORTC QLQ-C30, while the resting pressure was positively correlated with body image and sexual functioning domains of EORTC QLQ-CR38. On the basis of GHS scores suggestive of poor QoL and the correlation of urgency as well as manometrical findings, RST volume in particular, with QoL scores and no alteration in manometrical findings with respect to urgency; anorectal dysfunction seems to translate in an impairment of the QoL scores in our cohort.

Sphincter-saving surgery is generally preferable to APE among patients with rectal cancers given that it enables restored bowel continuity [6]. However, manometrical findings were suggestive of significant bowel dysfunction, while Wexner scores indicated severe fecal incontinence and poor scores were noted on functional and symptom domains of cancer specific and colorectal cancer specific QLQ in our cohort. Hence, our findings indicate long-term adverse effects of nCRT and sphincter-preserving surgery on bowel function and QoL in rectal cancer patients. Given the likelihood of overall QoL scores to be similar in patients with a stoma compared to those with re-continuity [6, 55], our findings emphasize that maintaining an adequate functional sphincter is more important than preserving the anal sphincter itself in terms of avoiding the gastrointestinal late effects including fecal incontinence and improving the patients’ QoL. Besides, given that nCRT has only limited benefit on overall survival, but a detrimental effect on function, we agree with the suggestion that the selection of patients for neoadjuvant therapy should be more conservative [38].

Hence our findings emphasize the implementation of pre-treatment counseling to inform patients of the risk of bowel dysfunction, routine postoperative screening for bowel dysfunction along with a need for more individually targeted follow-up and support in rectal cancer patients and increased awareness about not only oncological but also long-term functional outcome among surgeons [6, 38, 47].

Certain limitations to this study should be considered. First, due to single center design of the present study, establishing the temporality between cause and effect as well as generalizing our findings to overall rectal cancer population seems difficult. Second, relatively low sample size might prevent us to achieve the statistical significance concerning the change in study parameters with respect to tumor location. Third, while functional assessments were performed after the first postoperative year in almost 80 % of our patients, due to retrospective design of the study, lack postoperative duration prior to functional assessments was not standard and varied between patients. This may affect the results of functional assessments. Nevertheless, despite these certain limitations, given the paucity of the solid information available on this area, our findings represent a valuable contribution to the literature.

## Conclusions

In conclusion, our findings indicate significant late adverse effects of nCRT and sphincter preserving surgery on bowel dysfunction and QoL, particularly in the concomitant presence of urgency symptom, in rectal cancer patients. Given the association of manometrical findings on anorectal dysfunction with QoL, our findings emphasize the importance of manometrical evaluation in patients undergoing transient ileostomy following LAR operation prior to the stomal closure. Hence, identifying the patients with a high risk of developing functional anal impairment as well as the systematic registration of not only oncological but also functional and QoL related outcome seem important in all patients in the long-term disease follow-up. Further investigation is necessary to develop comprehensive assessment models for identification and careful selection of patients who are good candidates for restorative operations which would yield a baseline reference for new treatment modalities in patients who are at risk of experiencing late adverse effects of treatment.
